# Combined analysis of genome-wide expression and copy number profiles to identify key altered genomic regions in cancer

**DOI:** 10.1186/1471-2164-13-S5-S5

**Published:** 2012-10-19

**Authors:** Celia Fontanillo, Sara Aibar, Jose Manuel Sanchez-Santos, Javier De Las Rivas

**Affiliations:** 1 Cancer Research Center (CIC-IBMCC), Consejo Superior de Investigaciones Científicas (CSIC), Campus Miguel de Unamuno, Salamanca, Spain; 2 Department of Statistics, University of Salamanca (USAL), Salamanca, Spain

## Abstract

**Background:**

Analysis of DNA copy number alterations and gene expression changes in human samples have been used to find potential target genes in complex diseases. Recent studies have combined these two types of data using different strategies, but focusing on finding gene-based relationships. However, it has been proposed that these data can be used to identify key genomic regions, which may enclose causal genes under the assumption that disease-associated gene expression changes are caused by genomic alterations.

**Results:**

Following this proposal, we undertake a new integrative analysis of genome-wide expression and copy number datasets. The analysis is based on the combined location of both types of signals along the genome. Our approach takes into account the genomic location in the copy number (CN) analysis and also in the gene expression (GE) analysis. To achieve this we apply a segmentation algorithm to both types of data using paired samples. Then, we perform a correlation analysis and a frequency analysis of the gene loci in the segmented CN regions and the segmented GE regions; selecting in both cases the statistically significant loci. In this way, we find CN alterations that show strong correspondence with GE changes. We applied our method to a human dataset of 64 Glioblastoma Multiforme samples finding key loci and hotspots that correspond to major alterations previously described for this type of tumors.

**Conclusions:**

Identification of key altered genomic loci constitutes a first step to find the genes that drive the alteration in a malignant state. These driver genes can be found in regions that show high correlation in copy number alterations and expression changes.

## Background

Acquisition of somatic genetic alterations plays an important role in the development of cancer. Several systematic efforts have addressed the study of genetic alterations to characterize human cancers [1,2], including: copy-number alterations (CNAs), translocations, insertions or single-nucleotide polymorphisms (SNPs). Most of these approaches are focused on finding frequent alterations, which occur in a high number of cases. According to the *selective pressure *theory, a genomic alteration that confers an advantage to a malignant state is likely to be found in more tumors than expected by chance [3]. However, most methods that look for recurrent aberrations using copy number information find many regions, containing many genes [4,5]. Therefore, to identify recurrently altered genomic regions -biologically relevant- it is necessary to integrate gene and genome information, as proposed by Akavia *et al*. [3]. Several reports have recently shown that integrative strategies can be very useful to identify driver genes, considering the hypothesis that disease-associated gene expression changes are frequently induced by genomic alterations [3,6-10]. Most of these reports are focused on finding gene-based relationships.

Built on these hypotheses -that relate transcriptomic and genomic alterations-, we propose a new integrative method based on the location of both types of signals along the genome. Our method takes into account the genomic loci, both in the copy number (CN) analysis and also in the gene expression (GE) analysis, and applies the segmentation step proposed by Ortiz-Estevez *et al*. [11]. These authors designed a method for robust comparison between CN and GE using paired samples. Such approach is based on a search for correlation between segmented CN regions and segmented GE regions to find the most significant simultaneous alterations. We follow this approach introducing two new steps to asses the matching between CN and GE loci: (i) first, a signal correlation analysis; (ii) second, an alteration frequency analysis. Using these analyses we propose a set of significantly altered genomic regions in the studied pathological state. In order to show the performance and demonstrate the value of our method, we use a dataset of 64 Glioblastoma Multiforme (GBM) samples with paired measurements of GE and CN (taken from [7,8]).

## Results and discussion

The method is designed for combined analysis of datasets from two types of genome-wide arrays: DNA genomic microarrays and RNA expression microarrays. These arrays provide copy number and expression quantitative data, respectively. The analysis places both types of signals along the genome, taking into account the gene loci for the CN data and the GE data. The rationale of the method is to search for copy number alterations with a major influence in the expression levels of the genes encoded. As a distinctive element from other integrative approaches we do not consider only SNPs or genes individually. We take into account the gene loci following the strategy described in [11], that is based on the application of the same smoothing and segmentation algorithm to CN and GE in order to establish comparable regions. Once we get the smoothed segments, we perform two independent analyses for each gene loci: a signal correlation analysis and an alteration frequency analysis. (The workflow described in Materials and Methods, presented in last figure, illustrates the procedure of the method including these two independent analyses).

### Analysis of correlation between gene expression and copy number levels

**Figure 1:**
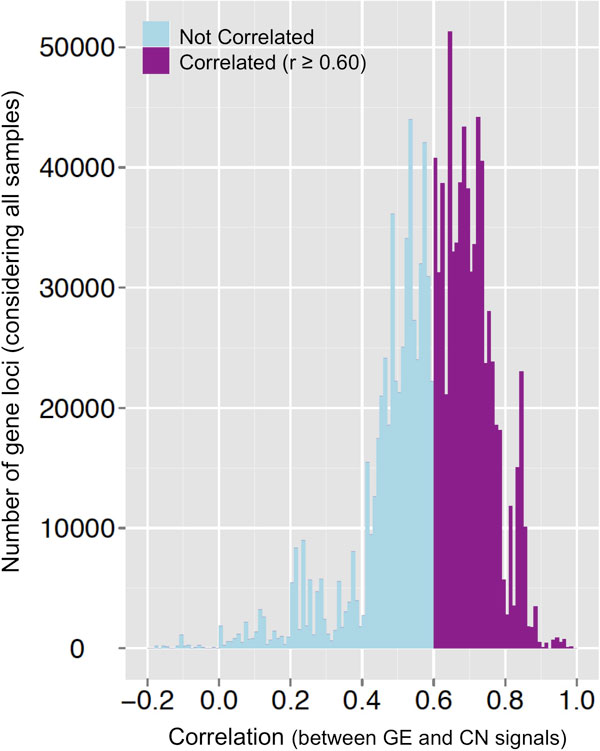
**Density distribution of the correlation coefficients between GE and CN for the GBM dataset**. Purple represents the number of gene loci that present significant correlation (r ≥ 0.60) between gene expression and copy number signals, counted considering all the samples. Blue are the rest, not considered significant.

The method matches the CN and GE segmented signals within each chromosomal region -i.e., the log2 ratio signals of the corresponding segments- and selects the gene loci that show a significant correlation. These loci can be considered candidate hotspots. In Figure 1 we present the results of this analysis done for the GBM dataset, marking in purple the number of gene loci with *Pearson *Correlation Coefficient r ≥ 0.60 (that corresponds to a Bonferroni-adjusted p-value < 0.005). Such cutoff (r ≥ 0.60) includes around 55 % of the human gene loci, providing a good coverage with a significant p-value. Setting more stringent cutoffs reduces the coverage too much: r ≥ 0.70 includes only ~26 % of the gene loci; r ≥ 0.80 includes only ~6 %.

The number of probes in the SNP arrays -used to calculate the segmented signals for CN- is large and uniform along the genome. However, in the expression arrays some genomic regions do not have enough allocated gene loci and the number of probes is sparse. This fact is a problem when a GE segment includes outliers (i.e. gene locus which have expression levels very different from the mean of their neighbours). To solve this problem, we look for statistically significant outliers within the GE segments -which were at least in 1/3 of the samples- and we recalculate the signal correlation between their unsegmented GE and the corresponding CN segments. In this way, we find a new set of gene loci with correlation r ≥ 0.60, which is added to the initial set of candidate hotspots identified. This step of the procedure is important to recover some gene loci with quite significant correlation (e.g. EGFR or SEC61G), which were missed in the first step due to the described problem.

### Analysis of frequencies for the categorical states Up-Gain and Down-Loss

**Figure 2:**
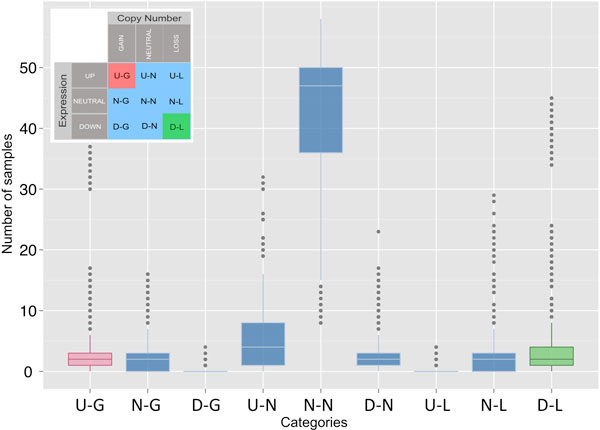
**Observed frequency distributions of the 9 categorical states for the GBM dataset: U-G, N-G, D-G, U-N, N-N, D-N, U-L, N-L, D-L**. Boxplots corresponding to the distributions of number of samples assigned to each category for all the gene loci. Insert: Contingency table showing the 9 possible categories for the gene expression and copy number. The total number of GBM samples analysed was 64.

The method also proposes to find the genomic regions that present a significant GE and CN alteration in the same direction. To assess this, we included a second selective step based on stratification of the segmented data. The genomic regions are stratified in several categories: up-regulation (**U**), down-regulation (**D**) or no-change (**N**) for expression; and gain (**G**), loss (**L**) or no-change (**N**) for copy number. This approach allows a discretization of the genomic regions into 9 different categories as shown in Figure 2 (inserted table): **U-G**, **N-G**, **D-G**, **U-N**, **N-N**, **D-N**, **U-L**, **N-L**, **D-L**. Figure 2 also presents the empirical cumulative distributions for these 9 categories of the GBM samples per gene loci, counting the frequency of samples for all the gene loci in each category. As expected, the distributions show that the "no change" (i.e. N-N, neutral-neutral) is the most frequent state. The analysis of distributions also finds some regions that show a clear correspondence between GE and CN alterations: i.e. the scenario where GE up-regulation is observed co-located with a CN gain (**U-G **category) and the scenario where GE down-regulation is co-located with a CN loss (**D-L **category). Our interest focuses on these regions, since they are the ones altered in the same way in both types of data. The analysis of the empirical frequency distributions for the **U-G **and **D-L **categories allows identifying the frequency cutoffs that correspond to the 10% upper quantiles. These cutoffs were: 13 samples for **U-G **and 11 samples for **D-L **(out of 64 in GBM dataset). The set up of these thresholds identifies those genomic regions that are the most frequently assigned to such altered categories (**U-G **and **D-L**) in the studied dataset.

### Genome-wide identification of hotspots: candidate key genomic regions

Our method identifies candidate key regions that show high correlation between CN and GE and that are frequently altered in the same direction, in both types of signals. The overlapping between the regions with the most significant correlation and the ones with the highest frequencies of simultaneous alteration (CN and GE) along the genome, will constitute hotspots where putative driver genes are likely to be encoded.

**Figure 3:**
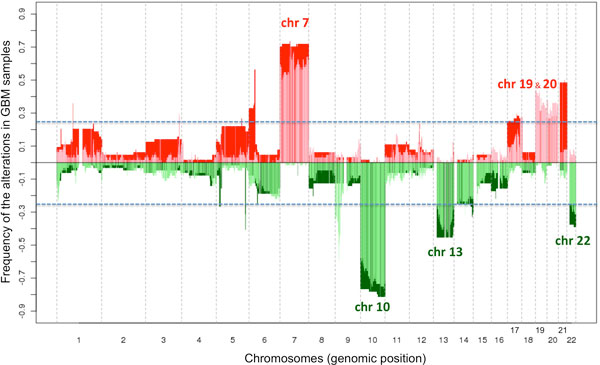
**Combined view of GE and CN alterations obtained for the GBM dataset**. The regions for all the human chromosomes (chr) that are altered either in CN or in GE are presented along the whole genome keeping the proportional size of the chromosomes. The graph shows the frequency of such alterations in the GBM samples. The colors correspond to GE up-regulated regions (in red) or down-regulated regions (in green), and -plotted on top- the CN gains (in pale red) or the CN losses (in pale green). Blue lines mark the regions that change in more than 25% of the GBM patients. The chromosomes with most significant changes (that present large regions included in the categories **U-G **or **D-L**) are labeled: **U-G **chr 7, chr 19, chr 20; **D-L **chr 10, chr 13, chr 22.

**Figure 4:**
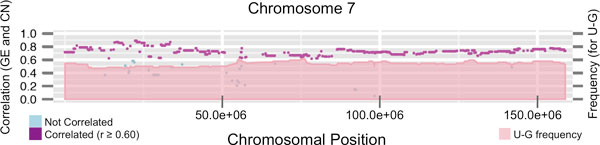
**Detailed view of chromosome 7 showing the CN and GE correlation and the U-G category frequency for the GBM dataset**. The genomic regions for chromosome 7 are represented in X-axis. Blue and purple dots show the correlation coefficients between CN and GE for each gene loci (purple when r ≥ 0.60). Pink profile represents the frequency values for the Up-Gain category **(U-G)**.

Figure 3 presents the combined view of GE and CN alterations on the complete genome obtained for the GBM dataset. The graph shows the alteration frequency, either in CN or in GE independently, along all the genome (22 human chromosomes). The dark colors correspond to GE up-regulated regions (red) or down-regulated regions (green), and the light colors -placed on top- correspond to CN gains (pale red) and losses (pale green). These results show that the method finds the alterations previously described for CN in GBM cancer [12,13]. In fact, the most frequent alterations in glioblastoma are the gain of chromosome 7 and the loss of chromosome 10. Our analysis finds such alterations in CN, and also finds their correlation with GE up-regulation for chromosome 7 and with GE down-regulation for chromosome 10. Figure 4 presents a detailed view of the alterations that occur in chromosome 7. It includes a profile of the regions with significant correlation (purple dots along the chromosome) and a profile of the frequency of **U-G **regions (pale red). They cover nearly the complete chromosome. A figure with the representation for all the 22 human chromosomes for the GBM samples is included as Additional File 3.

### Key genomic regions found for the 64 paired GBM cancer samples

As shown in Figure 3, the method presented in this work allows the identification of relevant altered genomic regions suffering significant changes in most of the GBM samples. The results also show that many of the detected CN alterations and GE changes overlap along the genome. These regions can be proposed as relevant "hotspots". In Table 1 and Table 2 we present a detailed description of the common genomic regions found in GBM; indicating the correlation and frequency of the **U-G **regions (Table 1, which includes 19 regions), and the **D-L **regions (Table 2, which includes 24 regions). The tables include the correlation between GE and CN for each region (as average correlation for all the gene loci); and the percentage of samples -frequency- in each region, counting only the samples where simultaneous GE and CN alterations occur: either up gene expression and gain in copy number (**U-G**) or down gene expression and loss in copy number (**D-L**). The regions detected are in the chromosomes that suffer the most significant changes in GBM samples: **U-G**, chr 7 and chr 20; **D-L **chr 10, chr 13, chr 14 and chr 22. The tables also include the genes enclosed in these regions. The most remarkable changes correspond to a large part of chr 7 (**U-G**) and to a large part of chr 10 (**D-L**). Two important genes are precisely located in these chromosomes: EGFR (in chr 7) usually up-regulated and PTEN (in chr 10) usually down-regulated [12,13]. PTEN is not found in our analysis, but it has been reported an absence of PTEN alterations in more than half of *de novo *glioblastomas and more than 90 % of glioblastomas developed from a pre-existing lower grade gliomas [14], which has been linked to the presence of additional tumor suppressor genes on chr 10, such as LGI1 [15] and MXI1 [16]. We found these two genes in regions 8 and 10 of the **D-L **list (Table 2), and we observed a very variable profile of PTEN in the GBM samples. These facts may indicate that PTEN is not the best genomic marker for this altered region. By contrast, we found RB1 tumor suppressor in region 13 of the **D-L **list; and this gene -included in chr 13- is a clear candidate to drive the alteration of tumor cells. With respect to EGFR, it has the highest **U-G **frequency observed (60.9%, Table 1) and therefore the method reveals this gene locus as the most common GE up-regulated and CN gained in the GBM samples. The alteration of EGFR can be associated with other genes that regulate its function, also found by the method. This is the case of VOPP1 and RAB11FIP2. VOPP1 is also known as ECOP (EGFR-coamplified and overexpressed protein) or GASP (Glioblastoma-amplified secreted protein), and is found in region 12 of the **U-G **list (Table 1). RAB11FIP2 is a suppressor of the endocytic internalization of EGFR and it is found in region 10 of the **D-L **list (Table 2) [17]. The presence of these genes in the hotspots found for GBM supports the value of the method described. There are many other interesting genes in the identified altered genomic regions, that can be useful for further investigations on the disease studied.

**Table 1:** Significant U-G regions with the associated genes.

regions	chr	cytobands	start	end	**Correlation **(average r coefficient)	**U-G Frequency **(average %)	Number of genes	gene symbols
1	**7**	p22.3,,p22.2,p22.1,...	13912	12407180	**0.75**	**53.13**	**97**	PDGFA,PRKAR1B,HEATR2,...
2	**7**	p21.2,p21.1	13980952	18581782	**0.83**	**48.34**	**16**	ETV1,DGKB,TMEM195,...
3	**7**	p21.1	19741898	19786077	**0.77**	**48.44**	**2**	TWISTNB,TMEM196
4	**7**	p21.1	20735744	20825207	**0.81**	**50.00**	**2**	ABCB5,SP8
5	**7**	p15.3,p15.2	22277336	26372745	**0.85**	**50.00**	**28**	RAPGEF5,IL6,TOMM7,...
6	**7**	p15.2	26682190	27829944	**0.68**	**50.00**	**18**	SKAP2,HOXA1,HOXA2,...
7	**7**	p14.3	29901392	33407268	**0.78**	**50.00**	**29**	WIPF3,SCRN1,FKBP14,...
8	**7**	p14.3,p14.2	34692740	36658380	**0.72**	**48.44**	**13**	NPSR1,DPY19L1,TBX20,...
9	**7**	p14.1,p13,p12.3,...	37856706	50759460	**0.72**	**49.60**	**83**	GPR141,TXNDC3,SFRP4,...
10	**7**	p11.2	54819940	54826939	0.77 *	**59.38**	**1**	SEC61G
11	**7**	p11.2	55086725	55275031	0.77 *	**60.94**	**1**	EGFR
12	**7**	p11.2	55572215	56043680	**0.70**	**59.06**	**5**	VOPP1,SEPT14,ZNF713,...
13	**7**	p11.2	56125502	56171766	**0.70**	**57.81**	**4**	CCT6A,SUMF2,PHKG1,...
14	**7**	p11.2,p11.1,q11.21	57269897	66582330	**0.66**	**56.56**	**25**	ERV3,VKORC1L1,GUSB,...
15	**7**	q11.22,q11.23,q21.11,...	69660980	91851882	**0.68**	**57.74**	**131**	AUTS2,WBSCR17,CALN1,...
16	**7**	q21.2,q21.3,	92738082	97975672	**0.72**	**56.51**	**36**	SAMD9,SAMD9L,HEPACAM2,...
17	**7**	q22.1,q22.3,q31.1,...	98456252	141707080	**0.71**	**55.94**	**343**	TMEM130,TRRAP,SMURF1,...
18	**7**	q34,q35,q36.1,...	141954920	158879258	**0.75**	**56.26**	**154**	TRBV12-2,TRBC1,PRSS1,...
19	**20**	p13,p12.3,p12.2,...	72762	62897316	**0.83**	**25.62**	**570**	DEFB125,DEFB126,DEFB12,...

Table with the significant Up-regulated and Gained (U-G) regions, indicating: chromosomal bands covered by each region; percentage of samples (%) that are in the U-G category in each region (calculated as average frequency for all the gene loci in the region); correlation between GE and CN for each region (calculated as average correlation of the gene loci in the region); number of genes located in each region. Total number of GBM samples analysed: N = 64. Marked with * the correlations calculated between unsegmented GE and segmented CN. Due to size limitations the table only includes a maximum of 3 cytobands or 3 genes. Complete information corresponding to this U-G regions is included as supplementary material: *Additional-file-*1.

**Table 2:** Significant D-L regions with the associated genes.

regions	chr	cytobands	start	end	**Correlation **(average r coefficient)	**U-G Frequency **(average %)	Number of genes	gene symbols
1	**10**	p14	9365826	11653762	**0.62**	**57.19**	**5**	CUGBP2,C10orf31,USP6NL,...
2	**10**	p13,p12.33,p12.31,...	16746068	24410224	**0.64**	**59.98**	**39**	RSU1,CUBN,TRDMT1,...
3	**10**	p12.1,p11.23,p11.22,...	25189544	47921720	**0.63**	**60.50**	**103**	PRTFDC1,ENKUR,THNSL1,...
4	**10**	p11.2	38238795	38265453	0.60 *	**60.94**	**1**	ZNF25
5	**10**	q11.22,,q11.23	49203737	53404614	**0.68**	**62.91**	**42**	FAM25C,BMS1P7,PTPN20C,...
6	**10**	q21.1,q21.2,q21.3,...	59989486	82351987	**0.65**	**64.07**	**152**	IPMK,CISD1,UBE2D1,...
7	**10**	q23.1,	84190870	87742781	**0.66**	**65.63**	**9**	NRG3,GHITM,PCDH21,...
8	**10**	q23.31,q23.32,q23.33	91498034	97024055	**0.62**	**67.19**	**39**	KIF20B,HTR7,RPP30,...
9	**10**	q24.1	97391080	97763109	**0.62**	**66.41**	**6**	ALDH18A1,TCTN3,ENTPD1,...
10	**10**	q24.1,q24.2,q24.31,...	98081203	134474152	**0.67**	**68.70**	**253**	DNTT,OPALIN,TLL2,...
11	**13**	q12.13,q12.2	27693163	28017254	**0.60**	**28.13**	**5**	USP12,RPL21,RASL11A,...
12	**13**	q12.2,,q12.3	28367434	30381664	**0.61**	**30.29**	**13**	GSX1,PDX1,ATP5EP2,...
13	**13**	q13.3,q14.11,q14.12,...	35881808	61059301	**0.73**	**35.48**	**124**	NBEA,MAB21L1,DCLK1,...
14	**13**	q21.32,q21.33	67340716	70478658	**0.62**	**35.94**	**2**	PCDH9,KLHL1
15	**13**	q22.2,q22.3,q31.1	76451567	80912598	**0.63**	**37.40**	**15**	KCTD12,IRG1,CLN5,...
16	**13**	q31.3,	92785210	94467454	**0.62**	**34.38**	**2**	GPC5,GPC6
17	**13**	q32.1,q32.2,q32.3,...	95812885	106130800	**0.64**	**33.35**	**38**	ABCC4,CLDN10,DZIP1,...
18	**13**	q33.3	108170700	108931486	**0.95**	**27.73**	**4**	FAM155A,LIG4,ABHD13,...
19	**13**	q34	110422550	111549152	**0.89**	**23.44**	**9**	IRS2,COL4A1,COL4A2,...
20	**14**	q11.2,q12,q13.1,...	19686156	70583360	**0.75**	**18.44**	**389**	TTC5,CCNB1IP1,PARP2,...
21	**14**	q24.2	71478110	72101080	**0.74**	**17.19**	**2**	PCNX,SIPA1L1
22	**14**	q24.2,,q24.3	73223406	76274521	**0.69**	**17.19**	**52**	DPF3,DCAF4,ZFYVE1,...
23	**14**	q24.3,,q31.1	77825772	80673424	**0.75**	**17.19**	**14**	TMED8,AHSA1,ISM2,...
24	**22**	q11.1,q11.21,q11.22,...	16157622	51224902	**0.73**	**24.93**	**490**	POTEH,CESK1,XKR3,...

Table with the significant Down-regulated and Lost (D-L) regions, indicating: chromosomal bands covered by each region; percentage of samples (%) that are in the D-L category in each region (calculated as average frequency for all the gene loci in the region); correlation between GE and CN for each region (calculated as average correlation of the gene loci in the region); number of genes located in each region. Total number of GBM samples analysed: N = 64. Marked with * : correlations calculated between unsegmented GE and segmented CN. Due to size limitations the table only includes a maximum of 3 cytobands or 3 genes. Complete information corresponding to this D-L regions is included as supplementary material: Additional-file-2.

Complete information corresponding to the genes found in the significant **U-G **regions and **D-L **regions is included respectively as supplementary material in **Additional-file-**1 (for the data corresponding to Table 1) and **Additional-file-**2 (for the data corresponding to Table 2).

## Conclusions

The combined analysis of CN and GE data obtained using DNA genome and RNA expression microarrays for paired samples is a very powerful approach to uncover key altered regions in a biological state studied. We present a robust method to find genomic regions that show simultaneous significant changes in both CN and GE. Our calculations applied to a cancer dataset find expected known genomic alterations and many others identified as key altered genomic regions. This approach is also proposed as an adequate strategy to identify driver or causal genes under the hypothesis that disease-associated gene expression changes are frequently induced by genomic alterations.

## Materials and methods

### Data

In this study we use a dataset of 64 human samples from Glioblastoma Multiforme (GBM) [7] that includes for each sample: *Affymetrix *DNA microarrays applied to detect of genome-wide CN changes and *Affymetrix *RNA expression microarrays applied to detect of GE changes. We used the same subgroup of samples that was previously analysed in Ortiz-Estevez *et al*. [11].

### GE and CN normalization and signals calculation

GE data were processed using RMA algorithm [18] applied to the human gene expression microarrays: *Affymetrix *HGU133 plus 2.0 (using the same strategy followed in [19,20]). CRMAv2 algorithm [21] was applied to normalize the raw data and obtain the signals from the *Affymetrix *Human Mapping 500K SNP arrays. The processed signals were divided by the median of the normal samples for each element (SNP or gene) and then the log2 was computed. These log2 ratio signals were smoothed and segmented using Circular Binary Segmentation (CBS) algorithm [22] with the default parameters implemented in the DNAcopy R package.

### Correlation between GE and CN

Pearson Correlation Coefficients (r) of the segmented GE and CN data were calculated taking the values of the segmented copy number and gene expression at the central point of the genomic position for each gene. P-values for the correlation coefficient of every gene loci were computed and adjusted by Bonferroni method. The established threshold for the selection of significantly correlated gene loci was correlation coefficient r ≥ 0.60, which corresponds to adjusted p-value < 0.005. When using the gene loci GE unsegmented signal, the same correlation threshold and p-value cutoff were applied.

### Frequency of U-G and D-L alterations

The thresholds that define DNA copy number gains and losses and up and down gene regulation were established applying k-Means algorithm, fixing three clusters (k = 3) on the segmented data, and done independently for the CN data and for the GE data. The CN data values were classified into gained (**G**), lost (**L**) or no-change (**N**) and the GE values were classified as up-regulated (**U**), down-regulated (**D**) or no-change (**N**). The thresholds found by k-Means for CN in the GBM dataset were > 0.19 (of the log2 ratio signals) for gain and < -0.15 for loss. The thresholds found for GE in the GBM were > 0.10 (of the log2 ratio signals) for up-regulation and < -0.12 for down-regulation. A contingency table with the 9 possible categorical states for the two types of data was built for every gene locus. A cutoff threshold was set up for the frequency of up-regulated and gained (**U-G**) and for the down-regulated and lost (**D-L**) categories, based on the empirical cumulative distributions of the categories. Taking into account the gene loci, the significant altered regions were defined as the ones that had a frequency ≥ than the upper 10% quantile of the distribution of **U-G **or the distribution of **D-L**.

### General workflow for identification of key regions in the genome

**Figure 5:**
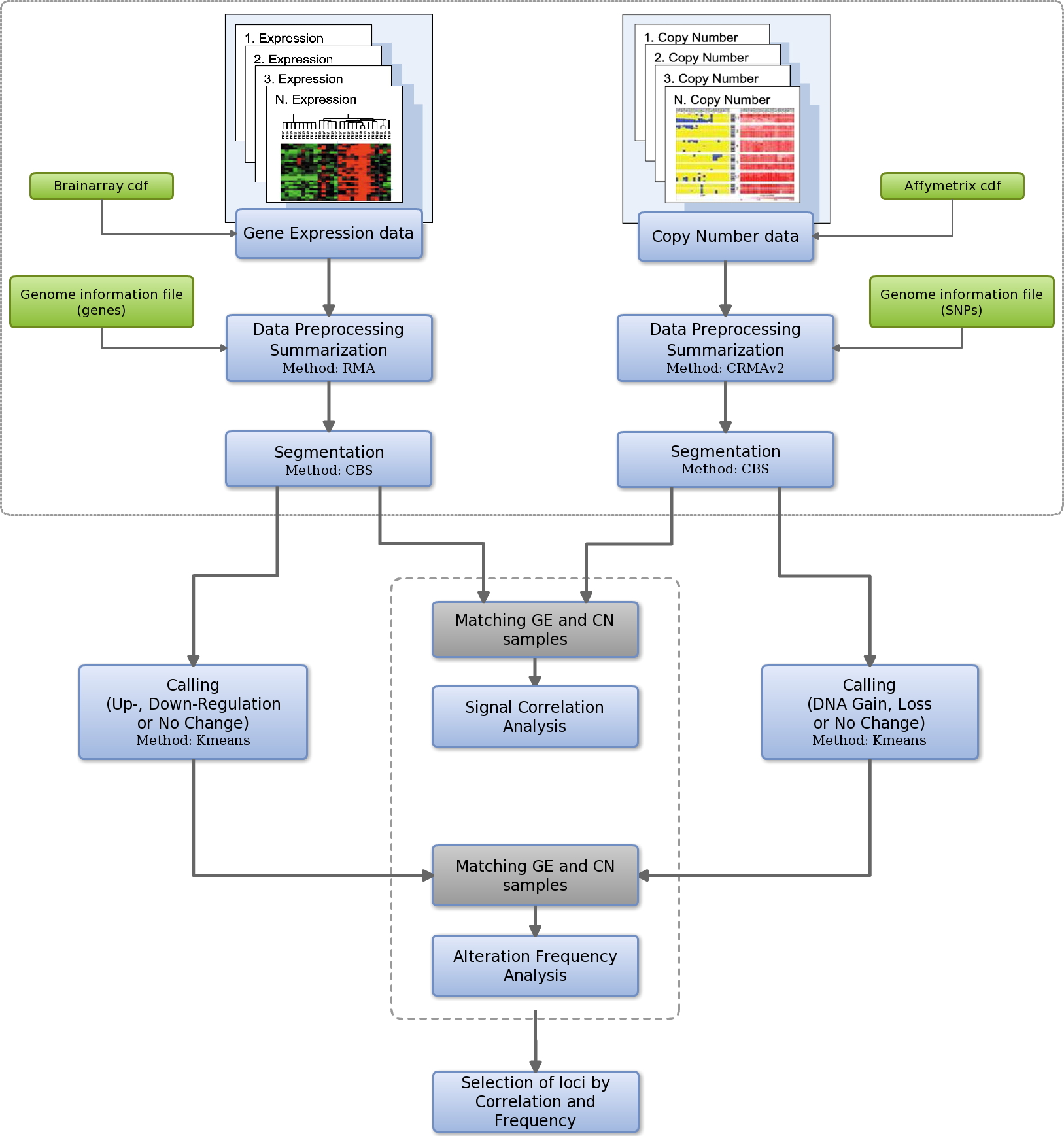
**Workflow of the method for the analysis of gene expression and copy number changes.** The figure illustrates the strategy to achieve a combined paired analysis of data sets from genome-wide microarrays both for GE and CN. The method searches for high correlation between segmented chromosomal regions that present a significant CN alteration (gain or loss) and segmented regions with significant GE change (up or down). The frequency of alteration is also analysed. The selected loci (hotspots) are regions chosen as significantly correlated and frequently altered in both GE and CN.

Following the steps described above, we present a general workflow (Figure 5) that illustrates the strategy to achieve a combined paired analysis of datasets from genome-wide microarrays, both for GE and CN. The workflow includes the different steps, the applied methods and the progression of the analysis. The strategy designed searches for high correlation between chromosomal regions that present a significant CN alteration (as gain or loss) and regions with significant GE change (as up or down). In this way, it determines which CN alterations have a strong influence on GE patterns. Key regions, i.e. hotspots in the genome, are defined as those regions simultaneously chosen as significantly correlated and frequently altered in both GE and CN.

## Competing interests

The authors declare that they have no competing interests.

## Authors' contributions

CF carried out most of the analyses, developed the proposed method and drafted the manuscript. SA helped in the computational analyses and in the presentation of the results. JMSS participated in the design of the study and in the statistical methods applied. JDLR conceived of the study, participated in its design and coordination and wrote the main manuscript. All authors read and approved the final manuscript.

## Supplementary Material

Additional file 1 **Spreadsheet with the complete data corresponding to Table **1. Click here for file

Additional file 2 **Spreadsheet with the complete data corresponding to Table **2. Click here for file

Additional file 3 **Detailed view of all the 22 chromosomes showing the CN and GE correlation and the U-G or D-L categories frequency for the GBM dataset**. The genomic regions are represented in X-axis. Blue and purple dots show the *correlation *coefficients between CN and GE for each gene loci (purple when r ≥ 0.60). Pink and green profiles represent the *frequency *values for the Up-Gain (U-G) category or the Down-Loss (D-L) category respectively. Click here for file
