# Supplementary figures and images for: Combined analysis of genome-wide expression and copy number profiles to identify key altered genomic regions in cancer
Source: BMC Genomics. 2012 Oct 19;13(Suppl 5):S5. doi: 10.1186/1471-2164-13-S5-S5 (PMC3476997; doi:10.1186/1471-2164-13-S5-S5)

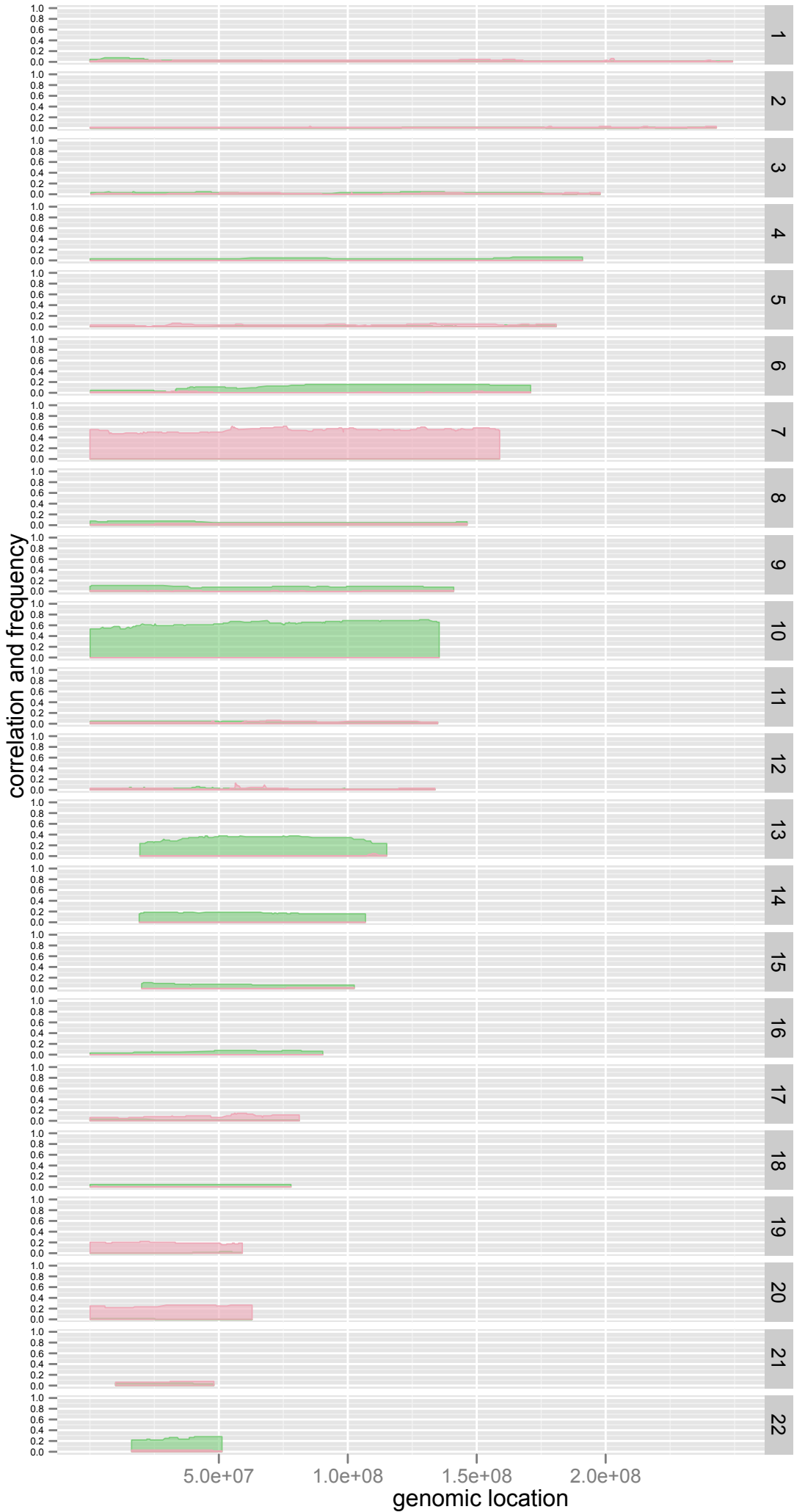

Supplement: Additional file 3 — Detailed view of all the 22 chromosomes showing the CN and GE correlation and the U-G or D-L categories frequency for the GBM dataset. The genomic regions are represented in X-axis. Blue and purple dots show the correlation coefficients between CN and GE for each gene loci (purple when r ≥ 0.60). Pink and green profiles represent the frequency values for the Up-Gain (U-G) category or the Down-Loss (D-L) category respectively. [file 1471-2164-13-S5-S5-S3.pdf]
